# Supplementary material for: Mechanistic modeling reveals a configurable AP-1 network governing cell-state heterogeneity and adaptive plasticity in melanoma cells
Source: Cell Rep. Author manuscript; Available in PMC 2026 Oct 12. (PMC13656306; doi:10.1016/j.celrep.2026.117965)
Supplement: Supplementary file 1 [file NIHMS2212235-supplement-1.pdf]

Cell Reports, Volume *45*

Supplemental information

Mechanistic modeling reveals a configurable AP-1 network governing cell-state heterogeneity and adaptive plasticity in melanoma cells

Yonatan N. Degefu, Magda Bujnowska, Douglas G. Baumann, and Mohammad Fallahi-Sichani

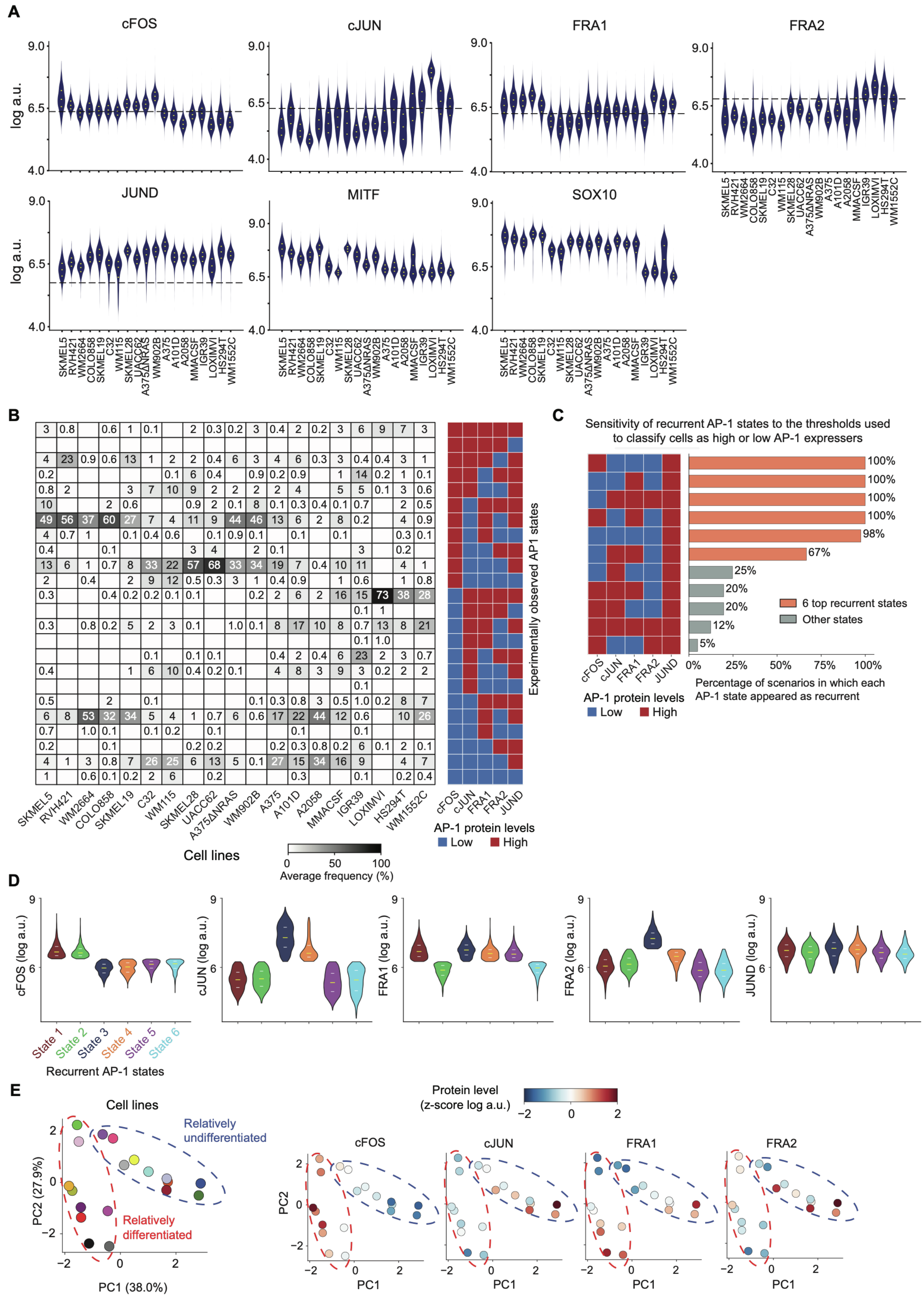

Figure S1. Melanoma cells express six recurrent AP-1 states linked to differentiation states across genetically diverse populations. Related to Figure 1. (A) Single-cell distributions of five AP-1 factors (cFOS, cJUN, FRA1, FRA2, JUND) and two differentiation state markers (MITF and SOX10) measured across 19 cell lines and shown by violin plots highlighting the median and

interquartile (25% and 75%) ranges. **(B)** Frequency of cells in each of the 24 experimentally observed AP-1 states (defined by the combinatorial expression of five AP-1 proteins) across 19 melanoma cell lines. Data are averaged across two biological replicates. **(C)** Sensitivity of recurrent AP-1 states to threshold selection. Recurrent AP-1 states were defined as those present at an average frequency >10% (across two biological replicates) in at least three of the 19 melanoma cell lines. To assess the robustness of state classification, binarization thresholds for each AP-1 protein were varied by ±0.15 natural log units, and recurrent states were re-identified across all threshold combinations, and percentage of scenarios in which each AP-1 state appeared as recurrent was shown. **(D)** Single-cell distributions of five AP-1 protein levels among the six recurrent AP-1 states combined across all cell lines. Violin plots show median and interquartile ranges. **(E)** Principal component analysis (PCA) of melanoma cell lines based on their single-cell frequencies in the six recurrent AP-1 states. Cell lines are colored either by identity (left) or by population-averaged expression of AP-1 proteins (right).

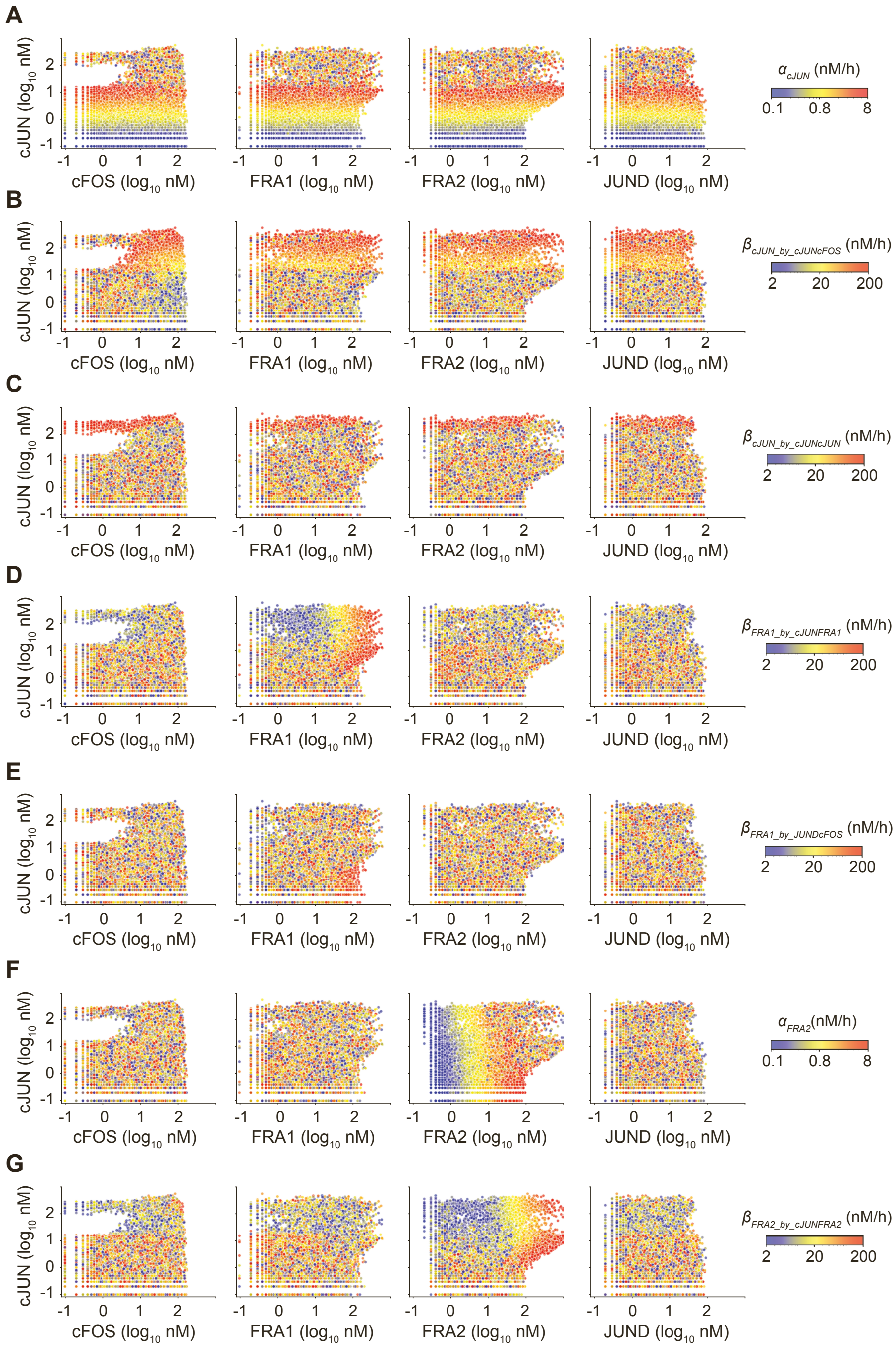

Figure S2. Visualization of AP-1 steady-state outcomes as a function of individual parameters. Related to Figure 2. Steady state outcomes from 4 million dynamic simulations, combining 200 initial conditions with 20,000 parameter sets sampled by Latin Hypercube Sampling (LHS), are shown. **(A-C)** Effects of basal production (A) or dimer-induced production of cJUN mediated by cJUN-cFOS homodimer (B) or cJUN-cJUN heterodimer (C). **(D, E)** Effects of dimer-induced production of FRA1 mediated by cJUN-FRA1 dimer (D) or JUND-cFOS dimer(E). **(F, G)** Effects of basal (F) or dimer-induced production of FRA2 mediated by cJUN-FRA2 dimer (G).

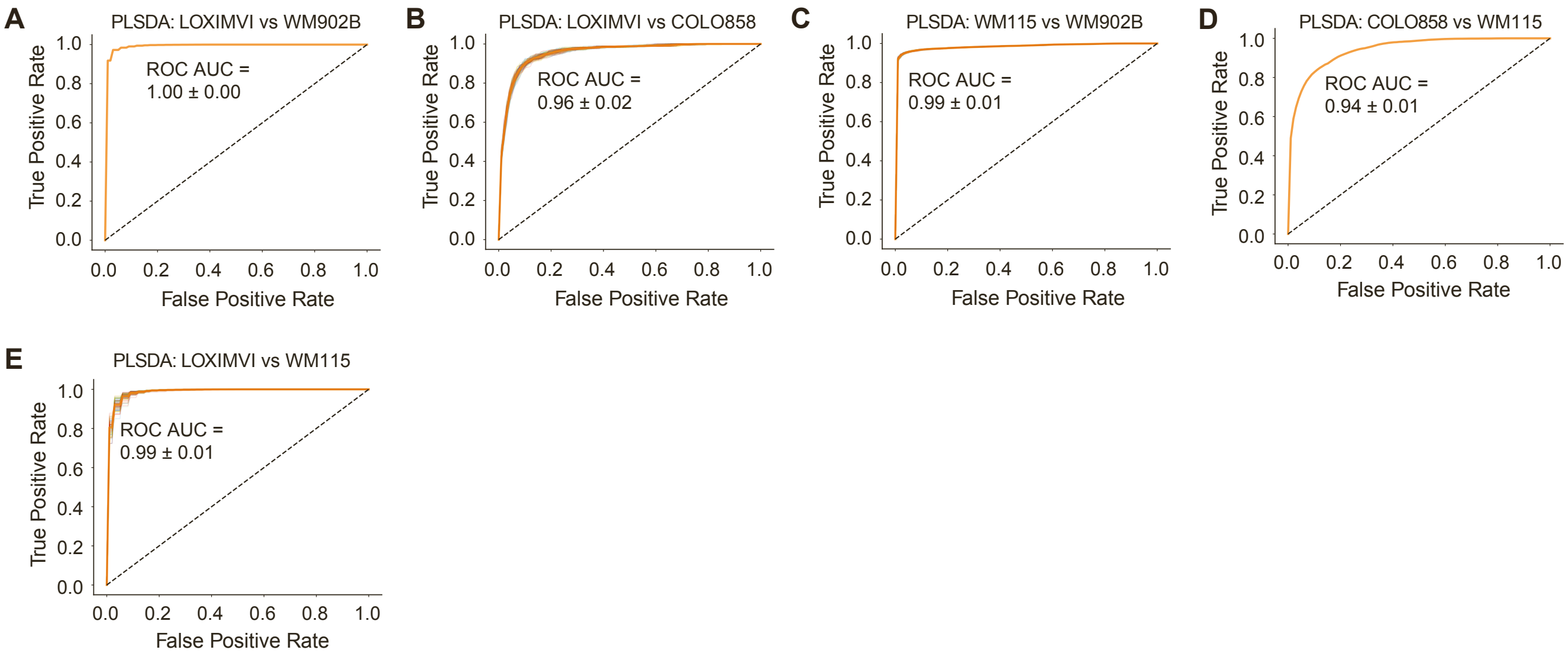

Figure S3. The area under the ROC curve shown as an overall measure of the PLS-DA classifier performance for pairwise comparisons across cell lines. Related to Figure 3. (A) PLS-DA model comparing cell populations from LOXIMVI versus WM902B. **(B)** PLS-DA model comparing cell populations from LOXIMVI versus COLO858. **(C)** PLS-DA model comparing cell populations from WM115 versus WM902B. **(D)** PLS-DA model comparing cell populations from COLO858 versus WM115. **(E)** PLS-DA model comparing cell populations from LOXIMVI versus WM115. Model performance was evaluated by five-fold stratified cross-validation, with the area under the ROC curve (AUC) reported as mean ± SD.

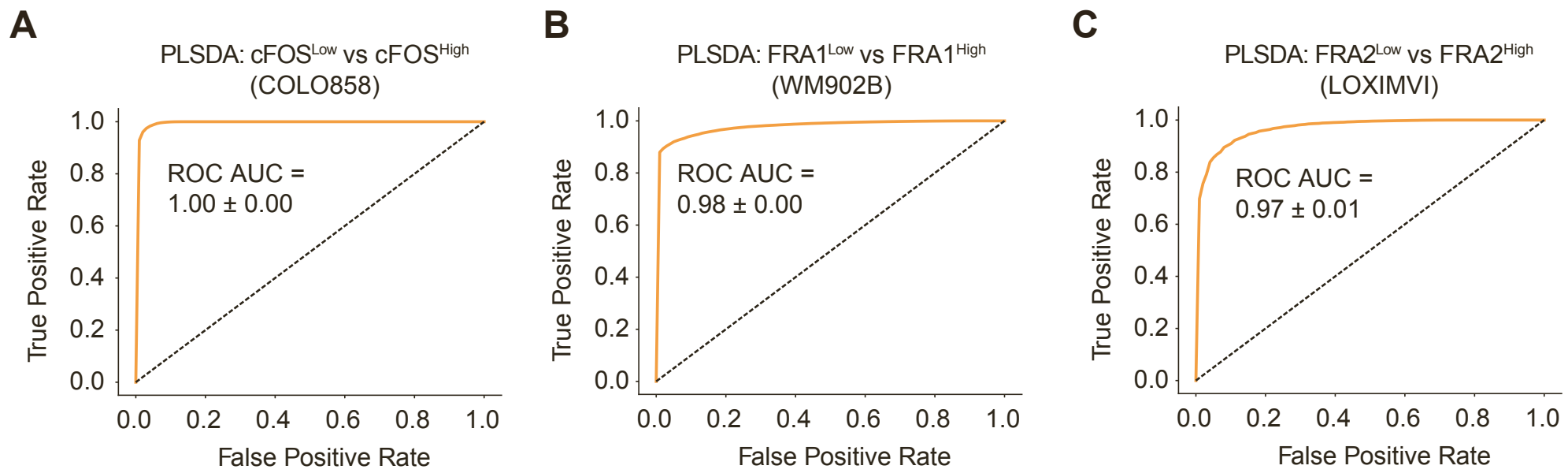

Figure S4. The area under the ROC curve shown as an overall measure of the PLS-DA classifier performance for comparison of cell subpopulations within cell lines. Related to Figure 4. (A) PLS-DA model comparing $cFOS^{High}$ and $cFOS^{Low}$ subpopulations in COLO858. **(B)** PLS-DA model comparing $FRA1^{High}$ and $FRA1^{Low}$ subpopulations in WM902B. **(C)** PLS-DA model comparing $FRA2^{High}$ and $FRA2^{Low}$ subpopulations in LOXIMVI. Model performance was evaluated by five-fold stratified cross-validation, with the area under the ROC curve (AUC) reported as mean ± SD.

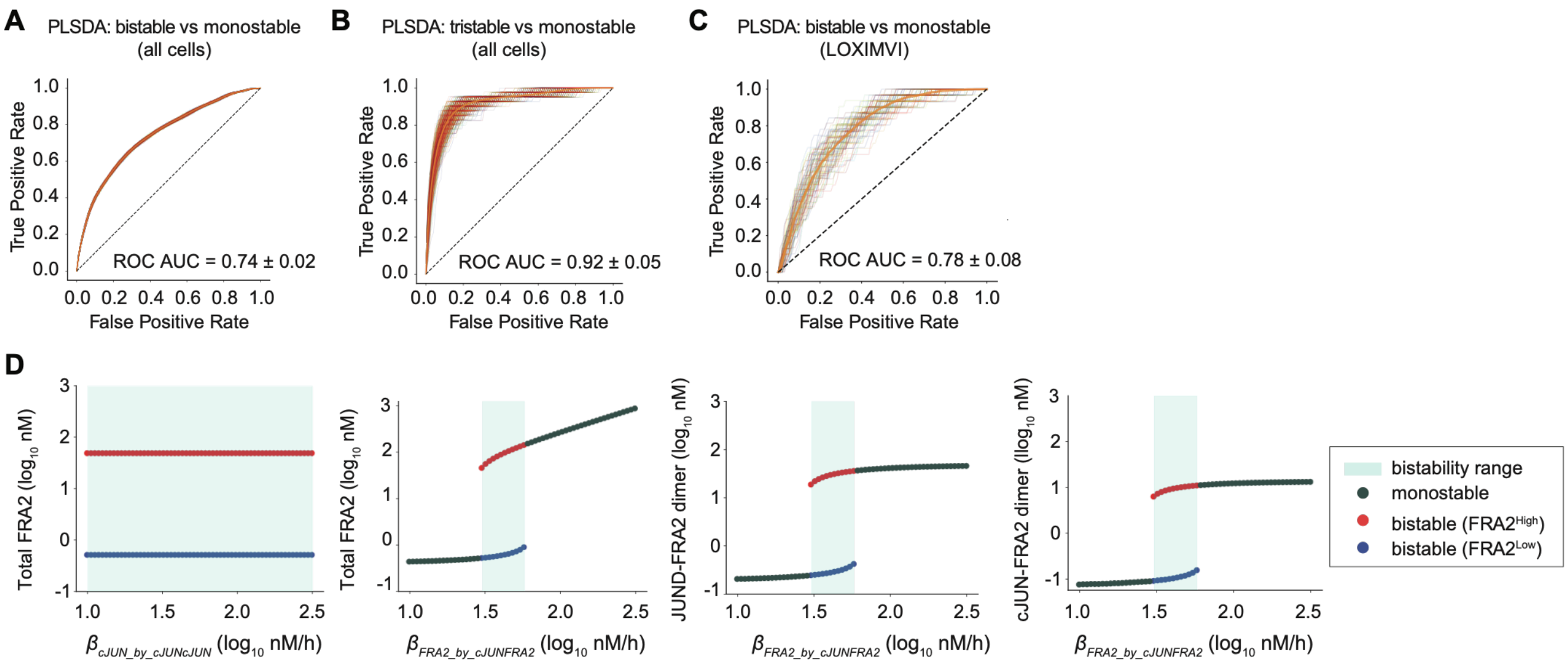

Figure S5. The balance between basal and dimer-induced FRA2 production determines FRA2 expression bistability. Related to Figure 5. (A-C) The area under the ROC curve shown as an overall measure of the PLS-DA classifier comparing bistable and monostable regimes among all cells (A), comparing tristable and monostable regimes among all cells (B), and comparing bistable and monostable regimes in LOXIMVI. Model performance was evaluated using five-fold stratified cross-validation, and the area under the ROC curve (AUC) is reported as mean ± SD. **(D)** Bifurcation diagrams showing the steady-state concentration of total FRA2 or FRA2-containing dimers (JUND-FRA2 and cJUN-FRA2) as a function of $\beta_{cJUN\_by\_cJUNcJUN}$ and $\beta_{FRA2\_by\_cJUNFRA2}$ for LOXIMVI cells. For each parameter, values were varied over a 60-fold range on a logarithmic scale while all other parameters were held fixed at their calibrated values. At each parameter value, the model was initialized using the corresponding set of initial conditions and simulated to steady state. Simulations were classified as monostable when all initial conditions converged to a single steady-state solution, and as bistable when initial conditions converged to two distinct steady-state solutions.

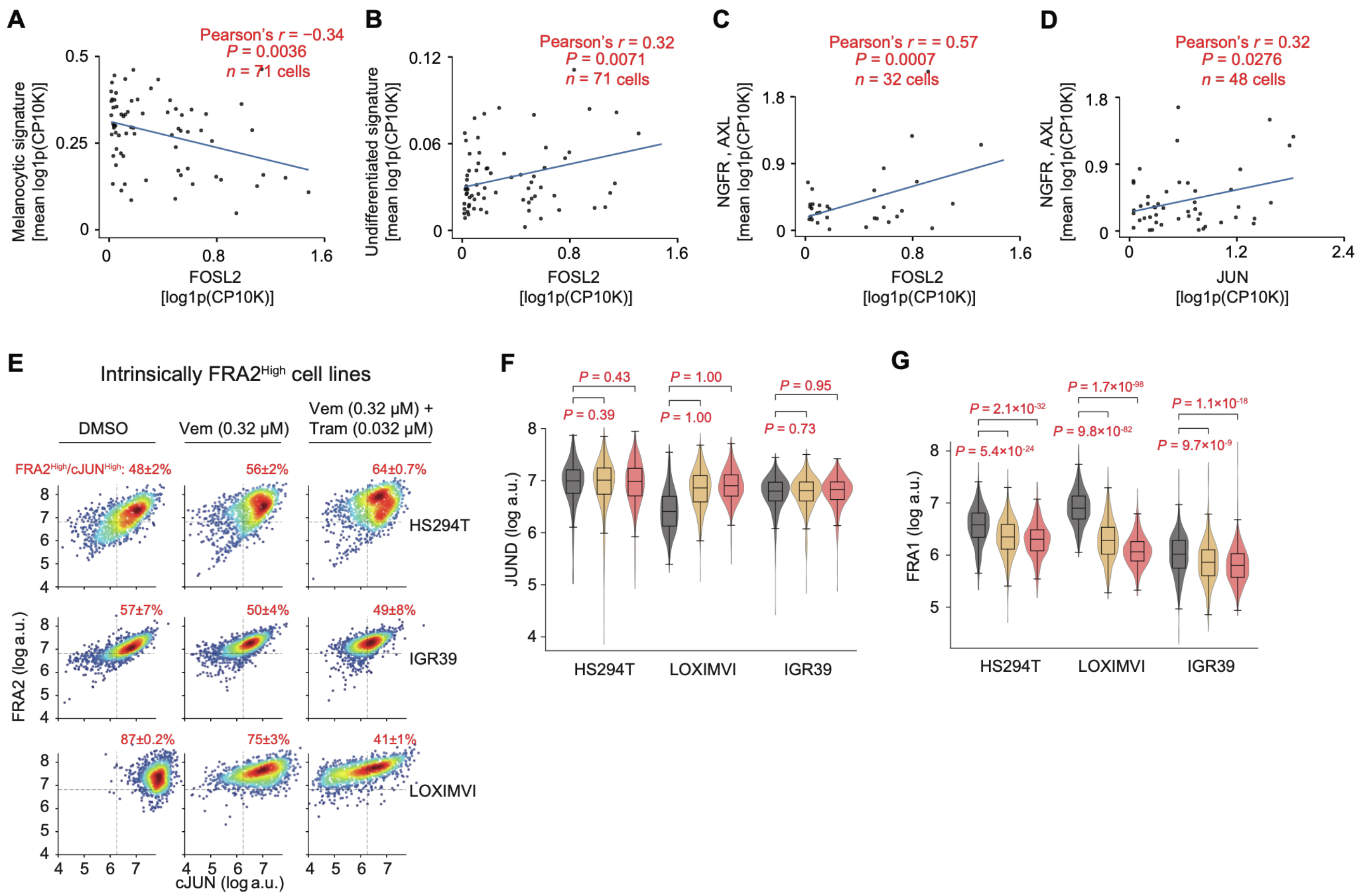

Figure S6. MAPK inhibitor-induced FRA2 upregulation is accompanied by cJUN upregulation, JUND downregulation, and FRA1 downregulation. Related to Figure 6. (A-C) Univariate associations between FOSL2 transcript abundance and the Tsoi et al.[1] melanocytic gene signature (A), undifferentiated gene signature (B), and undifferentiated melanoma markers NGFR+AXL (C). **(D)** Univariate association between JUN transcript abundance and undifferentiated melanoma markers (NGFR+AXL). Data are derived from single-cell RNA-seq analysis of a melanoma PDX dataset generated by Rambow et al.[2] Correlations were calculated using only cells with detectable expression of the AP-1 transcript under consideration. **(E)** Single-cell covariance analysis of FRA2 and cJUN protein levels in melanoma cells following 24 h treatment with Vemurafenib (0.32 µM), the combination of Vemurafenib (0.32 µM) and Trametinib (0.032 µM), or vehicle control (DMSO), measured by 4i. Data are shown for intrinsically $cJUN^{High}$/ $FRA2^{High}$ cell lines. The percentage of $cJUN^{High}$/ $FRA2^{High}$ cells (averaged across two replicates ± SD) is shown in the top-right quadrant of each plot. **(F,G)** Single-cell analysis of JUND (F) and FRA1 protein levels (G) in melanoma cells following 24 h treatment with Vemurafenib (0.32 µM), the combination of Vemurafenib (0.32 µM) and Trametinib (0.032 µM), or DMSO, measured by 4i. Data are shown for intrinsically $cJUN^{High}$/ $FRA2^{High}$ cell lines. Statistical comparisons were performed using one-sided Wilcoxon signed-rank tests. Box plots display medians and interquartile ranges.

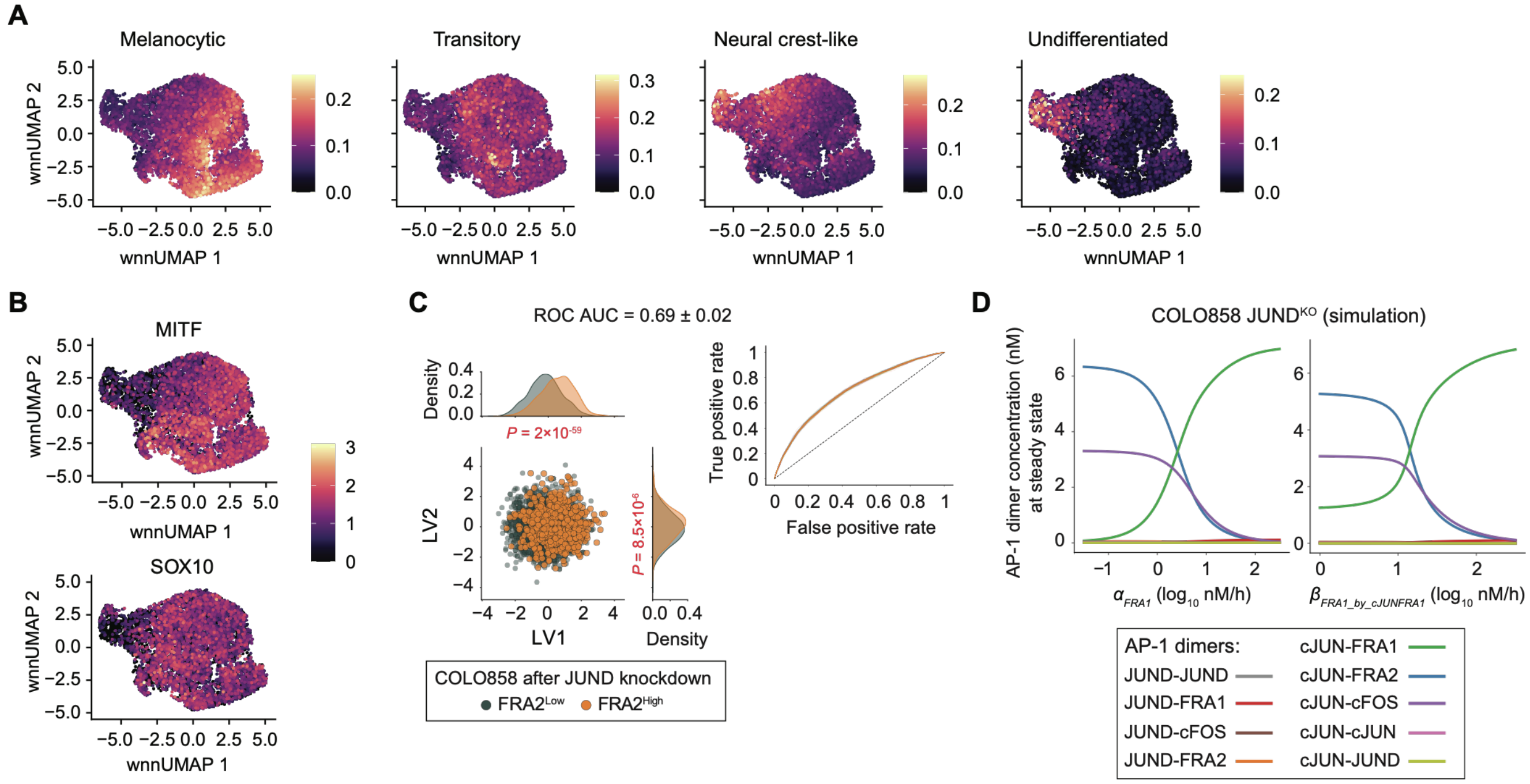

Figure S7. JUND loss promotes a FRA2^{High} state and drives an undifferentiated melanoma phenotype through reduced FRA1 and increased cJUN. Related to Figure 7. (A,B) Single-nucleus multiome (RNA + ATAC) analysis of COLO858 cells following JUND knockdown (JUND^{KD}) and in control conditions (NTC 1 and NTC 2) using weighted nearest-neighbor (WNN) integration. UMAP plots show WNN embeddings colored by Tsoi et al. differentiation-state signatures, including melanocytic, transitory, neural crest-like and undifferentiated states (A) or expression levels of MITF and SOX10 (B). **(C)** PLS-DA model comparing simulated FRA2^{High} and FRA2^{Low} COLO858 subpopulations following virtual JUND knockdown, using AP-1 network parameters (excluding those directly regulating FRA2) as input features. The PLS-DA score plot (left) shows individual cells colored by FRA2 expression state, with distributions compared across latent variables. Statistical significance along each latent variable was assessed using the Kolmogorov-Smirnov (KS) test. Model performance was evaluated using five-fold stratified cross-validation, and mean ± SD area under the ROC curve (AUC) is shown (right). **(D)** The impact of α_{FRA1} (left) and $\beta_{FRA1\_by\_cJUNFRA1}$ (right) on AP-1 network state under JUND depletion (JUND^{KO}) in COLO858 cells, assessed by a dose-response analysis via tracking model-predicted steady-state concentrations of AP-1 dimers across varying values of these parameters.

Table S1. Model parameters, their description, and estimated values or ranges used in this study. Related to STAR Methods

Parameter	Description	Unit	Value or Range	Source
α_{cFOS}	Basal production rate of cFOS	nM/h	0.08 - 80	Zhu et al.[3]
α_{cJUN}	Basal production rate of cJUN	nM/h	0.08 - 8	Zhu et al.[3]
α_{FRA1}	Basal production rate of FRA1	nM/h	0.08 - 8	Zhu et al.[3]
α_{FRA2}	Basal production rate of FRA2	nM/h	0.08 - 8	Zhu et al.[3]
α_{JUND}	Basal production rate of JUND	nM/h	0.08 - 8	Zhu et al.[3]
$\beta_{cJUN\_by\_cJUNcFOS}$	Production rate of cJUN induced by cJUN-cFOS dimer	nM/h	2 - 200	Zhu et al.[3]
$\beta_{FRA1\_by\_cJUNFRA1}$	Production rate of FRA1 induced by cJUN-FRA1 dimer	nM/h	2 - 200	Zhu et al.[3]
$\beta_{FRA2\_by\_cJUNFRA2}$	Production rate of FRA2 induced by cJUN-FRA2 dimer	nM/h	2 - 200	Zhu et al.[3]
$\beta_{cJUN\_by\_cJUNcJUN}$	Production rate of cJUN induced by cJUN-cJUN dimer	nM/h	2 - 200	Zhu et al.[3]
$\beta_{FRA1\_by\_JUNDcFOS}$	Production rate of FRA1 induced by JUND-cFOS dimer	nM/h	2 - 200	Zhu et al.[3]
γ_{cFOS}	Degradation rate of cFOS monomer	1/h	0.426 - 1.704	Basbous et al.[4]<br>Shah and Tyagi[5]
γ_{cJUN}	Degradation rate of cJUN monomer	1/h	0.417 - 1.668	Hernandez et al.[6]<br>Shah and Tyagi[5]
γ_{FRA1}	Degradation rate of FRA1 monomer	1/h	0.174 - 0.694	Basbous et al.[4]<br>Casalino et al.[7]
γ_{FRA2}	Degradation rate of FRA2 monomer	1/h	0.32 – 0.8	Alli et al.[8]
γ_{JUND}	Degradation rate of JUND monomer	1/h	0.058 - 0.232	Hernandez et al.[6]
$\gamma_{cJUNcFOS}$	Degradation rate of cJUN-cFOS dimer	1/h	$(\gamma_{cJUN} + \gamma_{cFOS}) / 2$	Estimated
$\gamma_{cJUNFRA1}$	Degradation rate of cJUN-FRA1 dimer	1/h	$(\gamma_{cJUN} + \gamma_{FRA1}) / 2$	Estimated
$\gamma_{cJUNFRA2}$	Degradation rate of cJUN-FRA2 dimer	1/h	$(\gamma_{cJUN} + \gamma_{FRA2}) / 2$	Estimated
$\gamma_{cJUNJUND}$	Degradation rate of cJUN-JUND dimer	1/h	$(\gamma_{cJUN} + \gamma_{JUND}) / 2$	Estimated
$\gamma_{cJUNcJUN}$	Degradation rate of cJUN-cJUN dimer	1/h	γ_{cJUN}	Estimated
$\gamma_{JUNDJUND}$	Degradation rate of JUND-JUND dimer	1/h	γ_{JUND}	Estimated
$\gamma_{JUNDcFOS}$	Degradation rate of JUND-cFOS dimer	1/h	$(\gamma_{JUND} + \gamma_{cFOS}) / 2$	Estimated
$\gamma_{JUNDFRA1}$	Degradation rate of JUND-FRA1 dimer	1/h	$(\gamma_{JUND} + \gamma_{FRA1}) / 2$	Estimated
$\gamma_{JUNDFRA2}$	Degradation rate of JUND-FRA2 dimer	1/h	$(\gamma_{JUND} + \gamma_{FRA2}) / 2$	Estimated
k_{off1}	Dimer dissociation rate between JUN and FOS family members	1/h	$k_{on1} \times KD_1$	Derived
KD_1	Dimer binding affinity between JUN and FOS family members	nM	2.8	Reinke et al.[9]
k_{on1}	Dimer association rate between JUN and FOS family members	1/(nM.h)	5.157	Kohler and Schepartz[10]<br>Wendt et al.[12]

k_{off2}	Dimer dissociation rate between JUN family members	1/h	$k_{on2} \times KD_2$	Derived
KD_2	Dimer binding affinity between JUN family members	nM	$100 \times KD_1$	Estimated; Grondin et al.[11]
k_{on2}	Dimer association rate between JUN family members	1/(nM.h)	5.157	Kohler and Schepartz[10];Wendt et al[12]
n	Hill Coefficient used to capture cooperative dimer binding to AP-1 promoter sites	dimensionless	1.5	Bintu et al.[13]<br>Ajo-Franklin et al.[14]
K_m	half-maximal activation constant for dimer-induced AP-1 production	nM	10	Bintu et al.[13]<br>Ajo-Franklin et al.[14]

Table S2. Edit-R synthetic crRNA and tracrRNA used for generating knockout cell lines. Related to STAR Methods

Oligonucleotide	Source, Cat#	Target sequence
**Edit-R Human JUN (3725) crRNA**	Horizon Discovery, Cat# CM-003268-01-0002	GTTGAGGGCATCGTCATAGA
**Edit-R Human JUND (3727) crRNA**	Horizon Discovery, Cat# CM-003900-01-0002	TAGAGGAACTGTGAGCTCGT
**Edit-R CRISPR-Cas9 Synthetic tracrRNA**	Horizon Discovery, Cat#, U-002005-05	
**Alt-R™ CRISPR-Cas9 tracrRNA, ATTO™ 550**	IDT, Cat# 1075927	

Table S3. Primers used for validation of knockout cell lines. Related to STAR Methods

Target gene	Forward primer	Reverse primer
**JUN**	GAGGTGAGGAGGTCCGAGTT	GCGTGCGCTCTTAGAGAAAC
**JUND**	CGGGAAGGGCACAGGTT	CGCCTCATCATCCAGTCCAA

References

1. Tsoi, J., Robert, L., Paraiso, K., Galvan, C., Sheu, K.M., Lay, J., Wong, D.J.L., Atefi, M., Shirazi, R., Wang, X., et al. (2018). Multi-stage Differentiation Defines Melanoma Subtypes with Differential Vulnerability to Drug-Induced Iron-Dependent Oxidative Stress. Cancer Cell *33*, 890-904.e5. https://doi.org/10.1016/j.ccell.2018.03.017.

2. Rambow, F., Rogiers, A., Marin-Bejar, O., Aibar, S., Femel, J., Dewaele, M., Karras, P., Brown, D., Chang, Y.H., Debiec-Rychter, M., et al. (2018). Toward Minimal Residual Disease-Directed Therapy in Melanoma. Cell *174*, 843-855.e19. https://doi.org/10.1016/j.cell.2018.06.025.

3. Zhu, R., del Rio-Salgado, J.M., Garcia-Ojalvo, J., and Elowitz, M.B. (2022). Synthetic multistability in mammalian cells. Science *375*, eabg9765. https://doi.org/10.1126/science.abg9765.

4. Basbous, J., Chalbos, D., Hipskind, R., Jariel-Encontre, I., and Piechaczyk, M. (2007). Ubiquitin-Independent Proteasomal Degradation of Fra-1 Is Antagonized by Erk1/2 Pathway-Mediated Phosphorylation of a Unique C-Terminal Destabilizer. Molecular and Cellular Biology *27*, 3936–3950. https://doi.org/10.1128/MCB.01776-06.

5. Shah, K., and Tyagi, S. (2013). Barriers to transmission of transcriptional noise in a c-fos c-jun pathway. Mol Syst Biol *9*, 687. https://doi.org/10.1038/msb.2013.45.

6. Hernandez, J.M., Floyd, D.H., Weilbaecher, K.N., Green, P.L., and Boris-Lawrie, K. (2008). Multiple facets of junD gene expression are atypical among AP-1 family members. Oncogene *27*, 4757–4767. https://doi.org/10.1038/onc.2008.120.

7. Casalino, L., De Cesare, D., and Verde, P. (2003). Accumulation of Fra-1 in ras-Transformed Cells Depends on Both Transcriptional Autoregulation and MEK-Dependent Posttranslational Stabilization. Molecular and Cellular Biology *23*, 4401–4415. https://doi.org/10.1128/MCB.23.12.4401-4415.2003.

8. Alli, N.S., Yang, E.C., Miyake, T., Aziz, A., Collins-Hooper, H., Patel, K., and McDermott, J.C. (2013). Signal-dependent fra-2 regulation in skeletal muscle reserve and satellite cells. Cell Death Dis *4*, e692–e692. https://doi.org/10.1038/cddis.2013.221.

9. Reinke, A.W., Baek, J., Ashenberg, O., and Keating, A.E. (2013). Networks of bZIP Protein-Protein Interactions Diversified Over a Billion Years of Evolution. Science *340*, 730–734. https://doi.org/10.1126/science.1233465.

10. Kohler, J.J., and Schepartz, A. (2001). Kinetic Studies of Fos·Jun·DNA Complex Formation: DNA Binding Prior to Dimerization. Biochemistry *40*, 130–142. https://doi.org/10.1021/bi001881p.

11. Grondin, B., Lefrancois, M., Tremblay, M., Saint-Denis, M., Haman, A., Waga, K., Bédard, A., Tenen, D.G., and Hoang, T. (2007). c-Jun Homodimers Can Function as a Context-Specific Coactivator. Molecular and Cellular Biology *27*, 2919–2933. https://doi.org/10.1128/MCB.00936-06.

12. Wendt, H., Berger, C., Baici, A., Thomas, R.M., and Bosshard, H.R. (1995). Kinetics of Folding of Leucine Zipper Domains. Biochemistry *34*, 4097–4107. https://doi.org/10.1021/bi00012a028.

13. Bintu, L., Buchler, N.E., Garcia, H.G., Gerland, U., Hwa, T., Kondev, J., Kuhlman, T., and Phillips, R. (2005). Transcriptional regulation by the numbers: applications. Current Opinion in Genetics & Development *15*, 125–135. https://doi.org/10.1016/j.gde.2005.02.006.

14. Ajo-Franklin, C.M., Drubin, D.A., Eskin, J.A., Gee, E.P.S., Landgraf, D., Phillips, I., and Silver, P.A. (2007). Rational design of memory in eukaryotic cells. Genes Dev. *21*, 2271–2276. https://doi.org/10.1101/gad.1586107.
